# The Leeds Evaluation of Efficacy of Detoxification Study (LEEDS) prisons project: a randomised controlled trial comparing dihydrocodeine and buprenorphine for opiate detoxification

**DOI:** 10.1186/1747-597X-4-1

**Published:** 2009-02-05

**Authors:** Laura Sheard, Nat MJ Wright, Hany G El-Sayeh, Clive E Adams, Ryan Li, Charlotte NE Tompkins

**Affiliations:** 1 Leeds Institute of Health Sciences, Charles Thackrah Building, 101 Clarendon Road, Leeds, LS2 9LJ, UK; 2 HMP Leeds and Leeds PCT based at Leeds Institute of Health Sciences, Charles Thackrah Building, 101 Clarendon Road, Leeds, LS2 9LJ, UK; 3 North Yorkshire and York Primary Care Trust, The Briary Wing, Harrogate District Hospital, Lancaster Park Road, Harrogate HG2 7SX, UK; 4 Mental Health Services Research, Division of Psychiatry, University of Nottingham Duncan MacMillan House, Portchester Road, Nottingham, NG3 6AA, UK; 5 Department of Mental Health Sciences, University College London Holborn Union Building, Highgate Hill, London, N19 5LW, England, UK

## Abstract

**Background:**

Many opiate users entering British prisons require prescribed medication to help them achieve abstinence. This commonly takes the form of a detoxification regime. Previously, a range of detoxification agents have been prescribed without a clear evidence base to recommend a drug of choice. There are few trials and very few in the prison setting. This study compares dihydrocodeine with buprenorphine.

**Methods:**

Open label, pragmatic, randomised controlled trial in a large remand prison in the North of England. Ninety adult male prisoners requesting an opiate detoxification were randomised to receive either daily sublingual buprenorphine or daily oral dihydrocodeine, given in the context of routine care. All participants gave written, informed consent. Reducing regimens were within a standard regimen of not more than 20 days and were at the discretion of the prescribing doctor. Primary outcome was abstinence from illicit opiates as indicated by a urine test at five days post detoxification. Secondary outcomes were collected during the detoxification period and then at one, three and six months post detoxification. Analysis was undertaken using relative risk tests for categorical data and unpaired t-tests for continuous data.

**Results:**

64% of those approached took part in the study. 63 men (70%) gave a urine sample at five days post detoxification. At the completion of detoxification, by intention to treat analysis, a higher proportion of people allocated to buprenorphine provided a urine sample negative for opiates (abstinent) compared with those who received dihydrocodeine (57% vs 35%, RR 1.61 CI 1.02–2.56). At the 1, 3 and 6 month follow-up points, there were no significant differences for urine samples negative for opiates between the two groups. Follow up rates were low for those participants who had subsequently been released into the community.

**Conclusion:**

These findings would suggest that dihydrocodeine should not be routinely used for detoxification from opiates in the prison setting. The high relapse rate amongst those achieving abstinence would suggest the need for an increased emphasis upon opiate maintenance programmes in the prison setting.

**Trial registration:**

Current Controlled Trials ISRCTN07752728

## Background

Prison populations include a high proportion of people who use illicit substances and are dependent upon illicit opiates [1]. Levels of drug use among prisoners tend to be much higher than in the general population [2]. In the United Kingdom (UK) almost two thirds of injecting drug users have spent some time in prison [3] and repeat drug-related offending and re-incarceration is common [3]. Illicit opiate users who enter the prison estate in the UK are typically offered pharmacological interventions of opiate maintenance treatment, or an opiate detoxification regime complimented by psychosocial support according to individual health need [4].

Historically, healthcare provision for injecting drug users in prisons in England and Wales has not been equivalent to that offered in community settings. There has recently been a phased re-organisation in prison healthcare with responsibility in England and Wales being transferred from the Home Office to individual National Health Service (NHS) Primary Care Trusts (PCT) [5]. Alongside this, current developments in drug policy have been designed to facilitate a change in prison based clinical practice to enable it to become equivalent to that offered in the community [6-9]. In support of this policy directive, financial resource is being provided to prisons to supply an integrated system for drug maintenance or detoxification treatment within nationally agreed clinical guidelines [4]. Consequently, all prisoners whose urine tests are positive for illicit opiates should be offered an opiate detoxification of equivalent standard to that in the community.

As many drug users reduce or cease illicit drug use whilst in prison, providing treatment for opiate detoxification is a core function of prison healthcare provision [4]. However, during the period when the current research was conducted (2004/2005) the evidence base and the national guidelines on the clinical management of drug misuse [10] did not stipulate a 'drug of choice' for opiate detoxification. In the absence of this evidence base, a wide variety of agents for opiate detoxification have previously been prescribed at the discretion of prison clinicians. Such medications include methadone, dihydrocodeine, buprenorphine, lofexidine and clonidine. Historically within UK prisons, the most commonly used drug for opiate detoxification has been dihydrocodeine. Anecdotally this was partly due to a reluctance to prescribe methadone following a small number of methadone related deaths in the prison setting. Dihydrocodeine has been attractive to clinicians as it has a shorter half-life than methadone, and seems equally acceptable to users. Robertson et al (2006) found that there was no significant difference in retention in treatment between dihydrocodeine and methadone for maintenance treatment in the community [11]. Towards the end of the study period (late 2005), there was a national move away from prescribing dihydrocodeine in the British prison setting due to its potential for diversion by prisoners into the shadow economy.

Buprenorphine, in the form of sub-lingual tablets, has the potential advantage of having a good safety profile, better retention in treatment and lower withdrawal severity when compared to methadone, lofexidine or clonidine [12-16]. Comparatively, it has been increasingly prescribed in the community for the purpose of opiate detoxification [17]. The latest Department of Health guidelines recommend either buprenorphine or methadone as first line agents for prisoners requesting an opiate detoxification, subject to clinician discretion [4].

Recently, the results of the Leeds Evaluation of Efficacy of Detoxification Study (LEEDS) were published [18]. The study was a randomised controlled trial (RCT) comparing buprenorphine and dihydrocodeine for opiate detoxification in the community drug treatment setting and showed that participants were more likely to achieve abstinence from illicit opiates at completion of detoxification with buprenorphine. However there are potentially differences in the demographics, drug histories and structuring of drug treatment in the community compared to the prison which limits the external validity of these findings in the prison treatment setting. Consequently, the research team considered it imperative that the same detoxification agents were compared within the prison estate. Additionally, there appears to be a paucity of clinical trials conducted worldwide in the prison setting which have evaluated medication for opiate detoxification. Whilst one British study evaluated the withdrawal severity of methadone versus lofexidine, the rates of completion were not sufficient to detect a statistically significant difference between the medications [19]. Given the dearth of randomised controlled trials for opiate detoxification in this environment, we felt it appropriate to introduce this methodology to answer an important research question which could inform clinical policy, decision making and prison policy directives [20]. This paper reports the findings of a trial comparing dihydrocodeine and buprenorphine for opiate detoxification in the UK prison setting.

## Methods

### Setting

Her Majesty's Prison Leeds. This is a large category B local remand prison^1 ^in the North of England, with over 1200 bed spaces. It accepts over 6000 adult male prisoners per year, primarily from the West Yorkshire area.

### Design

Pragmatic open label randomised controlled trial comparing two detoxification interventions – oral dihydrocodeine and sublingual buprenorphine. Randomisation sequence, with random block size, was generated using Microsoft Excel RAND function, by CEA in the Department of Psychiatry at the University of Leeds. CEA prepared sealed opaque consecutively numbered envelopes concealing the name of the allocated intervention. CEA had no contact with eligible participants.

The Multi Centre Research Ethics Committee for Wales (MREC Wales) approved the study in May 2004, and the Research Governance Organisation (Bradford South and West PCT) in April 2004.

### Eligibility

#### Inclusion criteria

1. Male (since research only took place in the male estate, thereby implicitly excluding women)

2. 18 – 65 years

3. Using illicit opiates as confirmed by a urine test taken at first assessment

4. Expressing a wish to detoxify through the standard monitored process and remain abstinent from opiates

5. Willing to give informed consent after receiving the participant information sheet

6. Remaining in custody in HMP Leeds for longer than 28 days

#### Exclusion criteria

1. Contraindications to dihydrocodeine or buprenorphine

2. Co-existing acute medical conditions requiring emergency admission for hospital care so precluding detoxification in the prison setting

3. Currently undergoing detoxification from other illicit drugs whereby concurrent detoxification from opiates would not be clinically indicated

4. Previous randomisation into the trial

### Recruitment

Participants were recruited from the medical reception area on arrival into HMP Leeds. On their first night in HMP Leeds, those with a current history of illicit opiate use (as confirmed by a Sure Screen multi panel drugs test) are routinely offered a detoxification regime. When prisoners who fulfilled the inclusion criteria approached the prison doctor, the purpose and rationale of the trial was explained to them. If they provided informed consent, the prison doctor (NW or HE) randomised them by opening the next pre-prepared opaque envelope and prescribing the intervention named within. Up to the point of opening the envelope both prisoner and doctor were blind to the intervention; once the envelope was open both prisoner and doctor knew the allocated intervention. On the opening of the envelope, the prison nursing staff and the prison pharmacist were informed of the allocated intervention for each participant. Standard clinical care continued from this point onwards.

Randomisation took place between July 2004 and July 2005. Some prisoners may have chosen to enter the trial as during this period, the standard detoxification choices offered to prisoners were dihydrocodeine and buprenorphine. This was regardless of whether they entered the trial or not, so it was only strong patient preference which predominantly meant people declined to take part. Recruitment was disappointing during the first five months of the trial (See Table 1 for breakdown of the actual rates of recruitment per month and also anticipated recruitment). So, in September 2004 (following MREC approval) the research team decided to provide an incentive of £5 which was credited to the prisoners' phone accounts (operated by a PIN number). The incentive was credited upon entry to the trial and prisoners were aware that they could withdraw at any time, yet keep the accredited incentive. We felt this incentive was an appropriate gratitude for prisoners to provide the voluntary urine samples and information that were required as part of taking part in the study. After introduction of the incentive, recruitment rates remained static but then increased in December and fluctuated somewhat. Ideally, it would have been beneficial to be able to provide incentives for urine samples at the secondary outcome points in order to increase the follow up rates.

**Table 1:** Rates of recruitment per month versus anticipated recruitment

	**Actual recruitment**	**Anticipated recruitment**
Jul 2004	1	10

Aug 2004	2	10

Sep 2004	2	10

Oct 2004	2	10

Nov 2004	0	10

Dec 2004	7	10

Jan 2005	19	10

Feb 2005	17	10

Mar 2005	13	10

Apr 2005	4	10

May 2005	12	10

Jun 2005	3	10

Jul 2005	8	10

### Interventions

Dihydrocodeine was given openly in the context of the standard prison doctor and drugs worker support. It was prescribed as a 30 mg oral tablet preparation "in-possession" medication. The medication was administered once a day to the participant who held the supply of medication to take in four daily divided doses.

Buprenorphine was given openly, in the context of the standard prison doctor and drugs worker support. It was dispensed either as 8 mg, 2 mg or 0.4 mg sublingual tablet preparation under daily supervised consumption.

The reducing regimen of both medications was at the discretion of the prescribing doctor. However, in practice, the detoxification regimes were subject to a protocol so as to fit into the high volume, busy nature of the prison regime. The dose prescribed did not exceed the standard regimes (Table 2). Therefore, the total dose administered was 96 mg of buprenorphine over 20 days and 6660 mg of dihydrocodeine over 20 days.

**Table 2:** Detoxification regimens

	**Buprenorphine**	**Dihydrocodeine**	
	
**Day**	**Dose (mg)**		
	
		morning	evening
**1**	6	2 × 120	2 × 120
**2**	8	2 × 120	2 × 120
**3**	8	2 × 120	2 × 120
**4**	8	2 × 120	2 × 120
**5**	8	2 × 120	2 × 120
**6**	8	2 × 120	2 × 120
**7**	8	2 × 120	2 × 120
**8**	8	1 × 120, 1 × 90	1 × 120, 1 × 90
**9**	6	1 × 120, 1 × 90	1 × 120, 1 × 90
**10**	6	2 × 90	2 × 90
**11**	4	2 × 90	2 × 90
**12**	3.6	1 × 90, 1 × 60	1 × 90, 1 × 60
**13**	3.2	1 × 90, 1 × 60	1 × 90, 1 × 60
**14**	2.8	2 × 60	2 × 60
**15**	2.4	2 × 60	2 × 60
**16**	2.0	1 × 60	2 × 60
**17**	1.6	1 × 60	2 × 60
**18**	1.2	1 × 60	1 × 60
**19**	0.8	1 × 60	1 × 60
**20**	0.4	XXXX	1 × 60

### Sample size

As no randomised controlled trials relevant to these comparisons have been previously undertaken in the prison setting, there was no comparable study on which to base the sample size calculation. The only other controlled trial comparing agents for detoxification (methadone and lofexidine) in a UK prison randomised 74 prisoners [19]. However, the project team completed a detoxification trial in the homeless community comparing dihydrocodeine with buprenorphine [20]. From this, we estimated that with a sample size of 60 we would have a finding of clinical and statistical significance for differences in the primary outcome. Due to loss of follow-up we determined a sample size of 120 would have sufficient power (i.e 80%) to determine a difference in the secondary outcomes between the two arms of 70% versus 45% [OR 1.56; α = 0.05 (two-sided)]. The power calculation was undertaken using Sample Power 1.20 developed by SPSS Inc., comparing two groups (60 individuals in each) and for α = 0.05 (two-sided).

### Data collection and Outcomes

The LEEDS trial co-ordinator (LS) collected details of allocated detoxification agent, background history, demographic details and use of opiates from the participant's prison medical records.

#### Primary outcome

Abstinence from illicit opiates at five days post detoxification as indicated by a supervised Sure Screen multi panel drugs test negative for opiates. This urine test was taken by a prison nurse who was prompted by LS at the appropriate follow-up time period.

#### Secondary outcomes

##### During the period of detoxification

*Serious and Adverse Events *– As part of routine clinical practice, clinicians and drugs workers noted any adverse events by making an entry in the participant's medical records. LS extracted data of adverse events clearly resulting in clinically significant distress to study participants or of major concern to clinicians from medical records, for the period of detoxification, and transcribed these onto a database.

*Leaving the study early *– perceived reasons for withdrawal were recorded.

*Inappropriate use of prescribed medication *– examples of this included intentional overdose, storing, trading, swapping or selling of prescribed medication.

*Service utilisation *– admission to hospital, Accident and Emergency and in-patient stays in prison hospital healthcare wing were recorded.

##### At 1, 3, and 6 months post detoxification

*Abstinence status *– if the prisoner was still in HMP Leedsthese data were extracted from clinical notes. If the person had been transferred, other prisons were contacted. If the prisoner had been released into the community, evidence of abstinence status was primarily ascertained through local community drugs service or GPs records. There was some, albeit limited, success tracing people via the address or telephone number which they had provided at the point of randomisation.

#### Service utilisation (as above)

All data was recorded on a Microsoft Excel spreadsheet.

Significant loss to follow-up occurred due to the high turnover of prisoners in HMP Leeds. Being a busy remand prison, the eligibility criteria of "remaining in custody in HMP Leeds for longer than 28 days" was determined so that the primary outcome would be complete for most participants. At the point of randomisation, remand prisoners who were due to appear in court in less than 28 days time were asked the likelihood of returning to HMP Leeds and invited to take part accordingly.

### Analysis

Following data entry, all analyses were undertaken using Review Manager 4.2.8 and SPSS software. The analysis of the primary outcomes was by a simple 2 × 2 table. Dummy tables were constructed for all secondary outcomes. These tables were designed as rigid templates for the final write up of the research, and to facilitate the researchers to collate a full data set and to mitigate against data dredging. Primary outcomes were analysed on an intention-to-treat basis: if the person did not finish the course of detoxification or did not give a urine sample then this was considered as a positive urine test for opiates. Intention-to-treat was used as a replication of the analysis performed in the trial conducted in the community [18] rather than other methods e.g. multiple imputation. Primary outcomes at follow-up were analysed both as per protocol (excluding those lost to follow-up) and intention-to-treat (missing urines assumed positive) with relative risk tests for categorical data and unpaired t-tests for continuous data. Secondary outcome data were analysed using chi square tests.

## Results

### Participants

**Figure 1:**
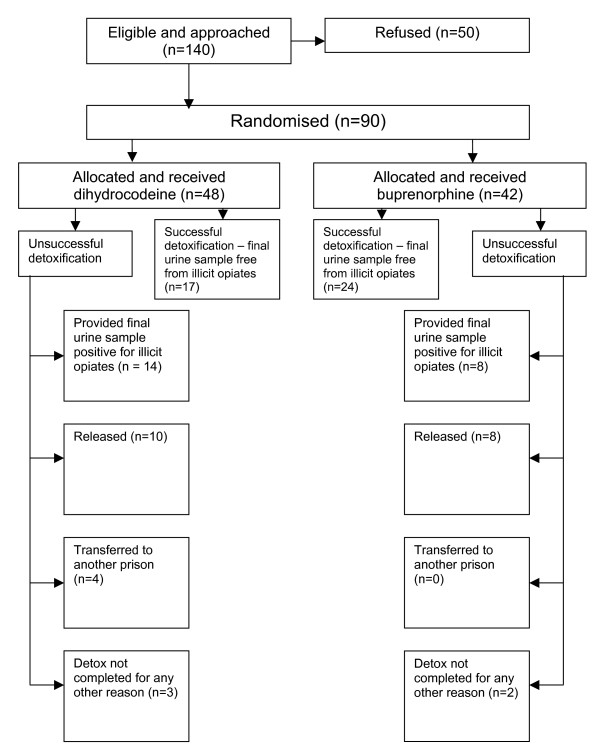
**Flow chart of randomisation outcome**.

Ninety men, that is 64% of those who were eligible and approached to take part in the study, consented to recruitment (Figure 1). These men were imprisoned in HMP Leeds and using illicit opiates prior to their sentence. The average age was 29.8 years (range 19–53) with the mean age for those allocated buprenorphine being 28.9 (SD 4.6) and 29.7 for those allocated dihydrocodeine (SD 6.1). The duration of using opiates overall was 9.3 years (range 1–18) with a mean of 8.9 years for those allocated buprenorphine (SD 3.5) and 9.7 years for those allocated dihydrocodeine (SD 4.6). Forty two men were randomly allocated to buprenorphine and 48 to dihydrocodeine. Variables relating to age, pattern of use and prognosis were evenly distributed between groups (Table 3).

**Table 3:** Demographic characteristics and prognostic factors

	**Buprenorphine** **(n = 42)**			**Dihydrocodeine** **(n = 48)**		
**Age **– mean (SD)	28.9 (4.6)			29.7 (6.1)		
**Pattern of use**						
**How are opiates taken?**						
IV	21 (50%)			18		
Smoking	9			12		
Methadone maintenance	2			0		
Don't know	10			18		
**Current daily use**						
**minimum **– mean (SD)	£40.00 (21.10)			£42.10 (29.40)		
**maximum**	£45.48 (23.39)			£45.65 (30.21)		
**Duration taking opiates **– mean (SD)	8.9 (3.5) years			9.70 (4.6) years		
**Initial urine**						
**illicit opiates present**	28/30			27/27		
**other drugs present**	16/30			13/27		
**Prognostic factors**						
	**Yes**	**No**	**D/K**	**Yes**	**No**	**D/K**
**Previous detoxes?**	27	3	4	25	4	3
**Successful detoxes?**	11	9	10	13	8	6

### Primary outcome

Overall, 63 men (70%) gave a urine sample at 5 days post detoxification (Table 4), between the two allocated regimens for provision of urine sample (RR 1.18, CI 0.90 – 1.54, z = 1.20, p = 0.43). 27 men (30%) did not provide a urine sample. 18 were released before their urine sample was due, 4 were transferred to another prison and 5 did not complete the prescribed detoxification regime. Of those released or transferred, 11 did not complete the detoxification, 4 completed the regime but left HMP Leeds before their urine test was due and 5 left on the day of the test or afterwards (data unavailable for 2). There was no statistically significant difference in demographic variables at baseline between those who completed detoxification and those who did not.

**Table 4:** Results

	**Buprenorphine** **(total = 42)**	**Dihydrocodeine** **(total = 48)**	**Relative Risk** **(95% CI)**	**Unpaired t-test**	**P value**
**By end of detox**					
**Final urine sample**	32/42	31/48	1.18 (0.90, 1.54)	N/A	0.43
**Per protocol negative urine**	24/32	17/31	1.37 (0.94, 1.99)	N/A	0.10
**ITT negative urine**	24/42	17/48	1.61 (1.02, 2.56)	N/A	0.04
**Leaving early**	10/42	15/48	0.76 (0.38, 1.51)	N/A	0.43
**Overdose**	0	0	N/A		
**Inappropriate use of allocated drug**	3/29	1/25	2.59 (0.29,23.32)	N/A	0.39
**A&E attendance**	0	0		N/A	
**Hospital admittance**	0	0		N/A	
**Prison doctor visits (mean)**	1.0 (0.5) n = 28	1.0 (0.6) n = 23	N/A	t(49) = 0.00	1.00

**At 1 months post detoxification**					

**Dead**	0/33	0/33		N/A	
**Abstinent (ITT)**	16/42	17/48	1.08 (0.63, 1.85)	N/A	0.79
**How known?**					
Urine test	4/10	2/5	1.00 (0.27, 3.72)	N/A	
Self report	12/21	15/25	0.95 (0.58, 1.55)	N/A	1.00
**A&E attendance**	0/33	0/33		N/A	0.84
**Hospital attendance**	0/33	0/33		N/A	
**Prison doctor visits (mean)**	0.4 (0.9) n = 25	0.6 (1.0) n = 28	N/A	t(51) = 0.76	0.45
**Drug worker visits** **(mean)**	0.2 (0.4) n = 3	2 n = 1		N/A	

**At 3 months post detoxification**					

**Dead**	0/27	0/23		N/A	
**Abstinent (ITT)**	13/42	12/48	1.24 (0.64, 2.41	N/A	0.53
**How known?**					
Urine test	2/8	1/4	1.00 (0.13, 8.00)	N/A	1.00
Self report	11/18	11/18	1.00 (0.59, 1.68)	N/A	1.00
**A&E attendance**	1/27	1/23	0.85 (0.06,12.87)	N/A	0.91
**Hospital attendance**	2/27	1/23	1.70 (0.16,17.60)	N/A	0.65
**Prison doctor visits (mean)**	0.8 (1.7) n = 17	1.5 (1.9) n = 17	N/A	t(32) = 0.76	0.27
**Drug worker visits (mean)**	0	No data		N/A	

***At 6 months post detoxification***					

**Dead**	0/14	0/12			
**Abstinent**	5/42	5/48	1.14 (0.36, 3.68)	N/A	0.82
**How known?**					
Urine test	1/3	1/4	1.33(0.13,13.74)	N/A	0.81
Self report	4/11	4/8	0.73 (0.26, 2.07)	N/A	0.55
**A&E attendance**	2/14	1/12	1.71(0.18,16.65)	N/A	0.64
**Hospital attendance**	3/14	2/12	1.29 (0.26, 6.46)	N/A	0.76
**Prison doctor visits (mean)**	2.0 (2.2) n = 4	2.2 (1.5) n = 4	N/A		
**Drug worker visits (mean)**	No data		N/A		

*ITT assumption = everybody not returning for final urine test had positive urine

* Statistical tests were z (approximation) tests

At the completion of detoxification, by intention to treat analysis, we found a higher proportion of people allocated to buprenorphine provided a urine sample negative for opiates (abstinent) compared with those who received dihydrocodeine (57% vs 35%, RR 1.61 CI 1.02–2.56, z = 2.065, p = 0.04).

### Secondary outcomes

At one month, follow up data were obtained on 66 participants (73% of the study sample). At three months, follow up data were obtained on 55 participants (61% of the study population). At six months, follow up data were obtained on 26 participants (29% of the study population).

At the 1, 3 and 6 month follow-up points, there were no statistically significant differences for urine samples negative for opiates between the two groups. There were also no statistically significant differences for any other of the secondary outcomes of Accident and Emergency attendance, hospital attendance, GP visits or drugs worker visits throughout this post-treatment period (Table 4). No serious adverse events were reported throughout the study.

## Discussion

### The findings

Our study showed that at five days after completion of the prison detoxification regime, buprenorphine at a total dose of 96 mg was more effective than dihydrocodeine at a total dose of 6660 mg in achieving abstinence from illicit opiates. It also showed that 43% of HMP Leeds' prisoners with a habit of illicit opiate use, who agreed to be included in the study, continued to acquire and use opiates even through the first few days of imprisonment and prescribed detoxification regimen.

It is possible that the research was underpowered to determine the effect of the interventions upon longer term abstinence, as at the secondary follow up points there were no statistically significant differences between the two groups for urine samples negative for opiates. However, it may be that there was clinically no significant difference. To this effect, firm conclusions regarding the effectiveness of buprenorphine and dihydrocodeine detoxification on post release opioid use and other core outcomes can not be conclusively determined. It is well known that post release from prison is a high risk time for relapse into illicit drug use. Therefore, it could be that more meaningful follow up data from prison based detoxification trials could be derived based on the time since release from prison in addition to time since detoxification.

A direct comparison can be made between the results of this study and those of the sister trial conducted in the community [18]. As previously stated, the result of the community trial also favoured buprenorphine over dihydrocodeine for opiate detoxification. Most importantly, completion of detoxification and provision of final urine in the prison environment was much higher than in the community (23% vs 70%). Reasons for this are varied but may include inherent characteristics of the treatment setting. For the participants who remained in HMP Leeds, when their urine sample was due, the closed, secure environment meant they were actively traced by prison nurses who took the urine samples. This contrasts with the very different environment of the community where the research team were dependent on the participants returning to their general practice to collect their final prescription so that a urine sample could be taken.

It is important to state that the trial did not introduce any new intervention medications into HMP Leeds as dihydrocodeine and buprenorphine were the only detoxification agents available during the period of randomisation [20]. The trial took place for a year from summer 2004, which is important as during this period the first line agent for most UK prisons was dihydrocodeine, with buprenorphine slowly being introduced. It seemed pertinent to compare these two agents, given that they were being prescribed to thousands of prisoners with a history of illicit opiate use every year in UK prisons despite no previous evaluation of their clinical effectiveness. Current policy recommendations are very different, with methadone and buprenorphine now being advocated as first line agents for opiate detoxification [4]. However, anecdotally, a practice of dihydrocodeine prescribing continues in many UK prisons.

Whilst there is a paucity of opiate detoxification trials conducted with prisoners, some studies have highlighted prisoners' subjective experiences of opiate detoxification. One recent qualitative study identified that prisoners in England who had been prescribed dihydrocodeine found that it was often inadequate at relieving acute opiate withdrawal and they were often reduced too quickly [21]. More favourable prison detoxification experiences were noted with buprenorphine and methadone [21]. Other studies have reported prisoners' sense of inadequacy in relation to short term methadone detoxifications [22,23] where the length of the detoxification is perceived as too short. Length of detoxification has now increased in UK prisons [4] and methadone and buprenorphine have become first line agents in the prison estate [4].

Current UK guidelines regarding the treatment of drug misuse in prisons recommend that only licensed opiate agonist medications (such as methadone or buprenorphine) should be used in the pharmacological treatment of opiate detoxification [4]. This recommendation was based on face validity consensus view of experts working in the field. Our findings strengthen and provide empirical support for the current guidelines which do not recommend the routine use of dihydrocodeine as a first line agent for detoxification in the prison setting. Outside of prison, recent clinical guidance from UK's National Institute of Clinical Excellence [24] has recommended against the routine use of dihydrocodeine for opiate detoxification based on evidence from the LEEDS trial in the community [18] and unpublished data from this current study. Both suggested no advantage in effectiveness of dihydrocodeine over buprenorphine either in the community or the prison.

### Methodological issues

LEEDS is only one of a small number of randomised controlled trials to take place in the UK prison estate. [25] The research team encountered barriers when conducting the community trial [18] such as patient preference, clinical equipoise and logistical issues [26]. Patient preference was a difficulty that carried through to the prison setting and was probably the largest hurdle to randomising people into the trial. However, conducting this trial in the prison environment presented many new problems and issues. Most significantly, the research team had to be satisfied that all prisoners gave informed consent and that they understood the processes of the trial. This was sometimes difficult in the noisy and chaotic environment of first night medical reception. Additionally, the reception area has a fast throughput and was not often conducive to the intricacies of a research trial. On some occasions, potential participants were not randomised if it was thought that they did not fully understand the concept of the trial or – more often – the process of randomisation. This was usually the case when they were in physical withdrawal from illicit opiates.

One weakness of the study was not recording demographic details of those who declined to participate as we cannot compare this group with those who agreed to be involved. Also this study involved men over the age of 18. Therefore applicability of the findings to women and young people in prison is problematic. Additionally, there are limitations with the intention-to-treat analysis used as this assumes that all missing urines tests are positive for opiates. Whilst we acknowledge this is problematic, this trial was analysed according to statistical convention in the UK and in keeping with the analysis of the sister trial previously conducted in the community.

Data collection was difficult when prisoners were transferred to serve their remaining sentence at other establishments across the wider prison estate. Indeed, there was a wide variation of responses to requests for help obtaining important information from other prison healthcare departments, despite having the necessary ethical and governance approvals in place to facilitate this. Whilst some prison healthcare departments at other establishments were willing to share information for the purposes of the trial, despite our best efforts, others refused. This sometimes led to a loss of follow up data from prisoners who were participating in the trial who had been transferred to certain unhelpful establishments.

As far as the research team are aware, this study is the only opiate detoxification randomised controlled trial in a prison setting which has taken abstinence from opiates (as indicated by a urine test) as the primary outcome. For studies assessing efficacy of opiate detoxification agents, an accurate and independent measure of abstinence status is important Cf [27]. The only other prison trial with which to compare is Howells et al (2002) [19]. In this UK study of 74 male prisoners with opiate addiction, lofexidine was compared with methadone with the primary outcome being self-reported withdrawal symptom severity during the detoxification period. Variously, other trials comparing agents for opiate detoxification in a variety of settings have used intensity/symptoms of withdrawal, retention in treatment, completion of treatment, nature of adverse effects [16,28] and relapse rate [28]. We believe that our first line method of ascertaining abstinence status via a urine test represents the most robust and binary manner in which to answer a clinical research question pertaining to the efficacy of detoxification medications.

We undertook this study on a minimal budget (one half time research assistant post for co-ordination and data collection over 19 months). Prison doctors (NW and HE) randomised in addition to their everyday clinical roles and responsibilities (approximately 5 to 8 minutes per participant). The research team believed this trial is imperative in order to ascertain whether a randomised controlled trial with drug using prisoners, which recorded abstinence, was feasible in UK prisons. The high throughput of large numbers of prisoners in an environment that is recognised as a high risk for overdose, self-harm and suicide [29] certainly presents significant logistical barriers to the smooth running of a research project. However, we do acknowledge that the very low follow up rate of this study is problematic and it may be pertinent for future studies to examine more rigorously whether buprenorphine was superior to dihydrocodeine at post release.

### Future research

This study raises further research questions. Since completion of this trial, the issue of buprenorphine abuse in the UK prison estate has been highlighted [30]. Therefore it could be that whilst our findings would suggest that dihydrocodeine should not routinely be used for detoxification in the prison setting, there could be other more effective agents than buprenorphine. In particular it is possible that methadone mixture is the pharmacological agent that is both most clinically effective and least amenable to diversion in the prison setting. Currently the Department of Health is supporting the research team to undertake a multi-prison trial comparing methadone with buprenorphine regimens for opiate detoxification. After a lengthy period completing the necessary approvals [31], recruitment for this trial began in January 2006. Randomisation and data collection for this research is currently ongoing, with almost 300 prisoners recruited to date across three prisons in the North of England. Methadone and buprenorphine are now the two first line detoxification medications within the British prison estate and a comparison is therefore fundamental to inform the current knowledge and evidence base.

The research team believe that conducting qualitative work around this trial may strengthen and give depth to the findings, particularly in relation to those people who were not abstinent at the primary outcome stage. It would have been interesting to understand how prisoners viewed the experience of their detoxification and how issues peculiar to the nature of prison life and drug use were worked out and overcome. Consequently, we would recommend that future randomised controlled trials (in a variety of settings) incorporate a qualitative element into their design in order to understand the holistic experience of simultaneously being a patient and research participant.

## Conclusion

This study suggests that buprenorphine may be more effective than dihydrocodeine for adult men undergoing opiate detoxification in the prison environment. However, it also demonstrates the high level of illicit opiate use within the prison estate even for those entered into a detoxification programme, with over 40% of prisoners in this study showing evidence of illicit opiates in their urine. The results of this trial reinforce current guidelines which do not recommend dihydrocodeine is prescribed as a first line agent for the management of opiate misuse. The high rate of relapse into opiate use post-intervention would also suggest a greater role for opiate maintenance in the prison estate as abstinence is not a realistic goal for many drug users within this environment. There is an emerging evidence base for the effectiveness of opiate maintenance programmes in the prison setting [32].

The research team encountered novel methodological issues and problems when randomising in the prison environment, the most crucial being informed consent. Data collection was also problematic once prisoners had been released or transferred to other establishments. In outlining our research experience, we hope to inform other research teams of the logistical issues of conducting a clinical trial in the British prison estate. Most significantly, this research demonstrates that a pragmatic randomised controlled trial *can *be undertaken in this difficult and challenging environment.

## Notes

1. Category B refers to a prison which is high but not maximum security. HMP Leeds is classed as a local prison in that it predominantly accepts men from the local area which is the county of West Yorkshire.

## Competing interests

The authors declare that they have no competing interests.

## Authors' contributions

NW and CA designed the study and offered project supervision. NW was principal investigator. CA centrally managed the randomisation process. LS co-ordinated and managed the project, assisted during randomisation clinics and collected follow up data. HE and NW randomised participants into the trial. RL conducted statistical analysis. All authors drafted the manuscript.
